# Joint Associations Between Body Mass Index and Waist Circumference With Atrial Fibrillation in Men and Women

**DOI:** 10.1161/JAHA.120.019025

**Published:** 2021-04-15

**Authors:** Michiel H. F. Poorthuis, Paul Sherliker, Gert J. de Borst, Jennifer L. Carter, Kin Bong Hubert Lam, Nicholas R. Jones, Alison Halliday, Sarah Lewington, Richard Bulbulia

**Affiliations:** ^1^ Clinical Trial Service Unit and Epidemiological Studies Unit Nuffield Department of Population Health University of Oxford United Kingdom; ^2^ Department of Vascular Surgery University Medical Center Utrecht Utrecht The Netherlands; ^3^ Medical Research Council Population Health Research Unit Nuffield Department of Population Health University of Oxford Oxford United Kingdom; ^4^ Nuffield Department of Primary Care Health Sciences University of Oxford Oxford United Kingdom; ^5^ Nuffield Department of Surgical Sciences University of Oxford Oxford United Kingdom; ^6^ Now with UKM Medical Molecular Biology Institute (UMBI) Universiti Kebangsaan Malaysia Kuala Lumpur Malaysia

**Keywords:** adiposity, atrial fibrillation, body mass index, sex‐specific risk factors, waist circumference, Atrial Fibrillation, Epidemiology, Ischemic Stroke, Obesity, Risk Factors

## Abstract

**Background:**

Associations between adiposity and atrial fibrillation (AF) might differ between sexes. We aimed to determine precise estimates of the risk of AF by body mass index (BMI) and waist circumference (WC) in men and women.

**Methods and Results:**

Between 2008 and 2013, over 3.2 million adults attended commercial screening clinics. Participants completed health questionnaires and underwent physical examination along with cardiovascular investigations, including an ECG. We excluded those with cardiovascular and cardiac disease. We used multivariable logistic regression and determined joint associations of BMI and WC and the risk of AF in men and women by comparing likelihood ratio χ^2^ statistics. Among 2.1 million included participants 12 067 (0.6%) had AF. A positive association between BMI per 5 kg/m^2^ increment and AF was observed, with an odds ratio of 1.65 (95% CI, 1.57–1.73) for men and 1.36 (95% CI, 1.30–1.42) for women among those with a BMI above 20 kg/m^2^. We found a positive association between AF and WC per 10 cm increment, with an odds ratio of 1.47 (95% CI, 1.36–1.60) for men and 1.37 (95% CI, 1.26–1.49) for women. Improvement of likelihood ratio χ^2^ was equal after adding BMI and WC to models with all participants. In men, WC showed stronger improvement of likelihood ratio χ^2^ than BMI (30% versus 23%). In women, BMI showed stronger improvement of likelihood ratio χ^2^ than WC (23% versus 12%).

**Conclusions:**

We found a positive association between BMI (above 20 kg/m2) and AF and between WC and AF in both men and women. BMI seems a more informative measure about risk of AF in women and WC seems more informative in men.

**Nonstandard Abbreviations and Acronyms**
WC: waist circumference

**Clinical Perspective**

**What Is New?**

- This large study describes, with unique reliability, the importance of obesity as a potentially modifiable risk factor for atrial fibrillation: body mass index being a more informative measure of atrial fibrillation risk in women and waist circumference in men.

**What Are the Clinical Implications?**

- The obesity epidemic sweeping across both high and low/middle income countries could drive up rates of atrial fibrillation and atrial fibrillation‐related strokes, and our findings make public health interventions to avoid weight gain increasingly pressing.

The prevalence of obesity has increased over recent decades, affecting over 2.5 billion people (almost 40% of the global population).^1^, ^2^ Individuals who are overweight or obese are at higher risk of cardiovascular disease, type 2 diabetes mellitus, cancer, and premature death.^3^, ^4^

Atrial fibrillation (AF) is the most frequent sustained cardiac arrhythmia in clinical practice and its prevalence is increasing.^5^ The estimated prevalence of AF in 2009 in the United States was 5.3 million of which 0.7 million were undiagnosed cases.^6^ The increasing burden of disease has been attributed mainly to aging populations but also to an increased AF incidence, related to the rise in prevalence of established AF risk factors such as hypertension and obesity.^7^ AF is associated with higher cardiovascular and cerebrovascular morbidity and mortality, including a 5‐fold higher risk of ischemic stroke.^8^ People with AF who are also overweight or obese are at even higher risk of ischemic stroke, thromboembolism, or death, compared with people with AF and healthy weight.^9^

Body mass index (BMI) has mainly been used to assess the relationship between adiposity measures and AF. Waist circumference (WC), a measure of abdominal or central adiposity, has received less attention than BMI yet may provide additional information on the risk of AF.^10^, ^11^, ^12^, ^13^, ^14^ Furthermore, whether the risk of AF varies across different adiposity measures and between sexes remains uncertain. For example, the association between WC and AF might differ across sexes as a result of differences in the distribution of adipose tissue. In this study, we used a large screened population to determine whether either BMI or WC alone or in combination better estimated the risk of AF risk in men and women.

## Methods

This study adhered to the STROBE (Strengthening the Reporting of Observational Studies in Epidemiology) statement (Table S1).

### Data Sharing

Data from large population‐based studies conducted by the Nuffield Department of Population Health can be shared with bona fide researchers on application to the principal investigators of this study. Details of the departmental data access policy can be found at https://www.ndph.ox.ac.uk/data‐access.

### Study Participants

This cross‐sectional study consisted of self‐referred and self‐funded individuals who attended a commercial vascular screening clinic between 2008 and 2013 in the United States and the United Kingdom.^15^ All participants completed an extensive questionnaire with information on their age, sex, height and weight, smoking status, alcohol use, history of diabetes mellitus, hypertension, vascular disease (coronary artery disease, stroke, transient ischemic attack, and peripheral arterial disease), congestive heart failure, valvular heart disease, left ventricular hypertrophy, and medication use (antiplatelet, antihypertensive, and lipid‐lowering medication).

BMI was calculated from self‐reported height and weight. Self‐reported anthropometric data showed to be suitable for use in analyses.^16^ We found a high correlation between reported height and measured height in a subset of 295 282 participants with a Spearman’s rho of 0.9461 (*P*<0.0001). We also found a high correlation between reported weight and measured weight in a subset of 292 176 participants with a Spearman’s rho of 0.9675 (*P*<0.0001). WC was measured by trained personnel using an inelastic tape measure. WC was defined as the smallest perimeter located between the last rib and the iliac crest, rounded to the nearest inch. Abdominal obesity was defined as WC of >102 cm in men or >88 cm in women.

In this study, we included 2 088 728 participants in whom BMI or WC was recorded and with ECG measurement, without a history of vascular disease (reported history of stroke, transient ischemic attack, coronary artery disease, or peripheral arterial disease), history of congestive heart failure, valvular heart disease, left ventricular hypertrophy, chronic obstructive pulmonary disease, or missing values for sex or smoking status (the full sample; Tables S2 and S3). Those with a history of vascular and cardiac disease were excluded to minimize reverse causation. BMI was available in 2 078 630 (99.5%) individuals and WC was available in 299 479 (14.3%) individuals BMI and WC were both recorded in 289 381 individuals. (Figure S1). Resurvey measurements for BMI were available for 8626 individuals rescreened at median 2.3 (interquartile range, 1.2–2.4) years later. Resurvey measurements for WC were available for 184 individuals rescreened at median 1.2 (interquartile range, 1.2–1.5) years later.

### Outcome and Its Ascertainment

The primary outcome was the prevalence of AF, measured with a single 12‐lead ECG. All ECGs were evaluated by physicians who received in‐house training.

### Statistical Analysis

BMI was categorized as follows: <20 kg/m^2^, 20 to <25 kg/m^2^, 25 to <30 kg/m^2^, 30 to <35 kg/m^2^, 35 to <40 kg/m^2^, and ≥40 kg/m^2^. WC was converted from inches to centimeters and categorized into quintiles. We calculated quintiles for men and women separately.

Baseline characteristics are presented as means and SD for continuous variables and as absolute numbers and percentages for categorical variables. Logistic regression models were used to estimate odds ratios (ORs) and 95% CI for AF. Models were adjusted for age at screening (with 5‐year intervals), sex, and country (“basic adjustment”) and additionally for smoking status (never, ever), alcohol use (never, 1–7 units, 8+units weekly), history of diabetes mellitus, history of hypertension, history of hypercholesterolemia, and use of antihypertensive medication and lipid‐lowering medication (“full adjustment”). We included 896 120 individuals in this multivariable model with full adjustment for BMI and the risk of AF, and 205 574 participants for WC and the risk of AF. Although some of these variables, like hypertension or cardiovascular medications, could be on the causal pathway between BMI and WC and the risk of AF, we controlled for them as confounders as we were interested in examining independent associations of these adiposity measures outside of these pathways.

For comparison of BMI and WC categories, the variance of the log odds in each group was calculated from the variances and covariances of the log ORs. This provides group‐specific CIs, which allow comparison between the BMI and WC categories without the choice of a reference group.^17^, ^18^ We also calculated ORs per 5 kg/m^2^ increment in BMI where the association was log‐linear (excluding the lowest BMI group). The ORs for WC were calculated for an equivalent multiple of the SD of BMI to facilitate the comparison between BMI and WC.

ORs were corrected for regression dilution using resurvey measurements for BMI and WC.^19^, ^20^ This correction accounts for measurement error and changes in BMI and WC between baseline and resurvey measures. ORs for each risk factor group were plotted against the mean of the resurvey values (ie, estimated “usual value”), and summary log ORs (and their SEs) were divided by the regression dilution ratio.^19^ The regression dilution ratios were calculated as Spearman self‐correlation regression dilution ratios (Table S4).

We compared the goodness‐of‐fit, using likelihood ratio (LR) χ^2^ statistics, to directly compare the associations between both BMI and WC and the risk of AF. These analyses were performed using the 193 140 participants in whom both BMI and WC were recorded, with BMI ≥20 kg/m^2^ and without missing values of covariates included in the multivariable model with full adjustment (the nested sample). The LR χ^2^ statistics were calculated as twice the increase in the log‐likelihood on the addition of extra terms of the logistic models after adding BMI and WC to the fully adjusted logistic model (without adiposity measures). With this we quantified the extent to which BMI and WC improve prediction of the prevalence of AF. We also compared the LR χ^2^ statistics of the logistic models after adding BMI to the fully adjusted logistic model with WC, and after adding WC to the logistic model with BMI to quantify the extent to which BMI and WC provide additional useful information.^21^ We performed these comparisons in all participants and in men and women separately.

We performed subgroup analyses by age, smoking status, alcohol use, history of diabetes mellitus, history of hypertension, or use of antihypertensive medication in participants in whom both BMI and WC were recorded (the nested sample).

STATA version 15.1 was used for statistical analyses and R version 3.5.1 was used for plotting figures.

### Ethical Approval

The University of Oxford Medical Sciences Inter‐Divisional Research Ethics Committee approved the study. All individuals consented for the data collected at the screening to be used for research purposes.

## Results

Baseline characteristics of 2 088 728 individuals are shown in Table 1 (full sample). The mean age was 63.6 (SD, 10.1), 65% were female, and ever smoking prevalence was 44% in men and 35% in women. A history of hypertension or use of antihypertensives was reported in 63% of the participants with AF and 46% of the participants without AF. A history of diabetes mellitus was reported in 17% of the participants with AF and 11% of the participants without AF. Mean BMI was 27.8 (SD, 5.3) kg/m^2^ in participants with BMI recorded and 28.7 (SD, 5.7) kg/m^2^ in 11 976 participants with AF. Mean WC was 94 (SD, 15.3) cm in participants with WC recorded and 103 (SD, 16.4) cm in 1521 participants with AF (Table 1). Mean BMI in participants in whom both BMI and WC was recorded was 28.2 (SD, 5.4) kg/m^2^. Baseline characteristics of participants with both BMI and WC recorded are provided in Table S5.

**Table 1:** Baseline Characteristics

	Participants With AF (N=12 067)	Participants Without AF (N=2 076 661)	All Participants (N=2 088 728)
Age, y	72.7±9.4	63.6±10.1	63.6±10.1
Female sex	4957 (41.1)	1 348 707 (64.9)	1 353 664 (64.8)
Height in men, m	1.79±0.1	1.78±0.1	1.78±0.1
Height in women, m	1.63±0.1	1.63±0.1	1.63±0.1
BMI, kg/m^2^ ^‡^	28.7±5.7	27.8±5.3	27.8±5.3
WC, cm^§^	102.6±16.4	94.1±15.3	94.1±15.3
Male sex ever smoker^||^	3598 (50.6)	320 997 (44.1)	324 595 (44.2)
Female sex ever smoker^||^	1635 (33)	474 811 (35.2)	476 446 (35.2)
Current alcohol use	2660 (44.8)	403 545 (43.2)	406 205 (43.2)
Hypertension or antihypertensive therapy	7070 (63)	877 658 (45.7)	884 728 (45.8)
Diabetes mellitus	1826 (16.6)	200 901 (10.5)	202 727 (10.6)
Hypercholesterolemia or lipid‐lowering therapy	5588 (51)	971 451 (50.7)	977 039 (50.7)
Creatinine, mg/dL^¶^	0.9±0.3	0.8±0.3	0.8±0.3

Values are mean±SD for continuous variables and n (%) for categorical variables. AF indicates atrial fibrillation.; BMI, body mass index; and WC, waist circumference.

^‡^ Mean BMI was 28.3±4.6 kg/m^2^ in all men, 29.0±5.2 kg/m^2^ in men with AF, and 28.3±4.6 kg/m^2^ in men without AF. Mean BMI was 27.6±5.6 kg/m^2^ in all women, 28.4±6.3 kg/m^2^ in women with AF, and 27.6±5.6 kg/m^2^ in women without AF.

^§^ Mean WC was 100.9±13.2 cm in all men, 105.9±14.9 cm in men with AF, and 100.8±13.1 cm in men without AF. Mean WC was 90.3±15.1 cm in all women, 96.9±17.2 cm in women with AF, and 90.3±15.1 cm in women without AF.

^||^ Ever smoker was defined as current or former smoker.

^¶^ Creatinine was measured in a subset of 92 534 participants.

**Figure 1:**
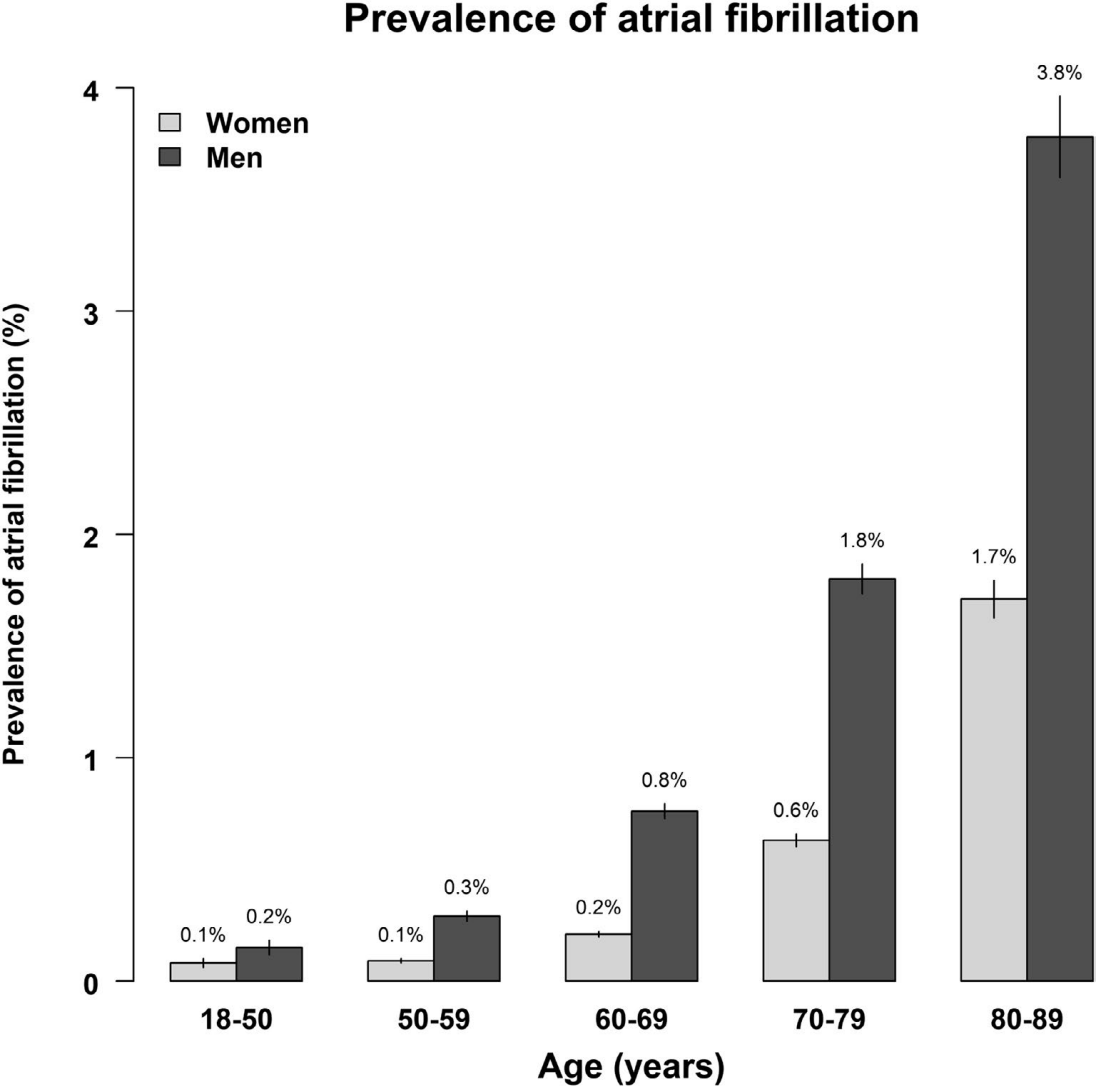
Prevalence of atrial fibrillation in men and women, by age. The vertical lines on the top of the bars represent the 95% CI.

**Figure 2:**
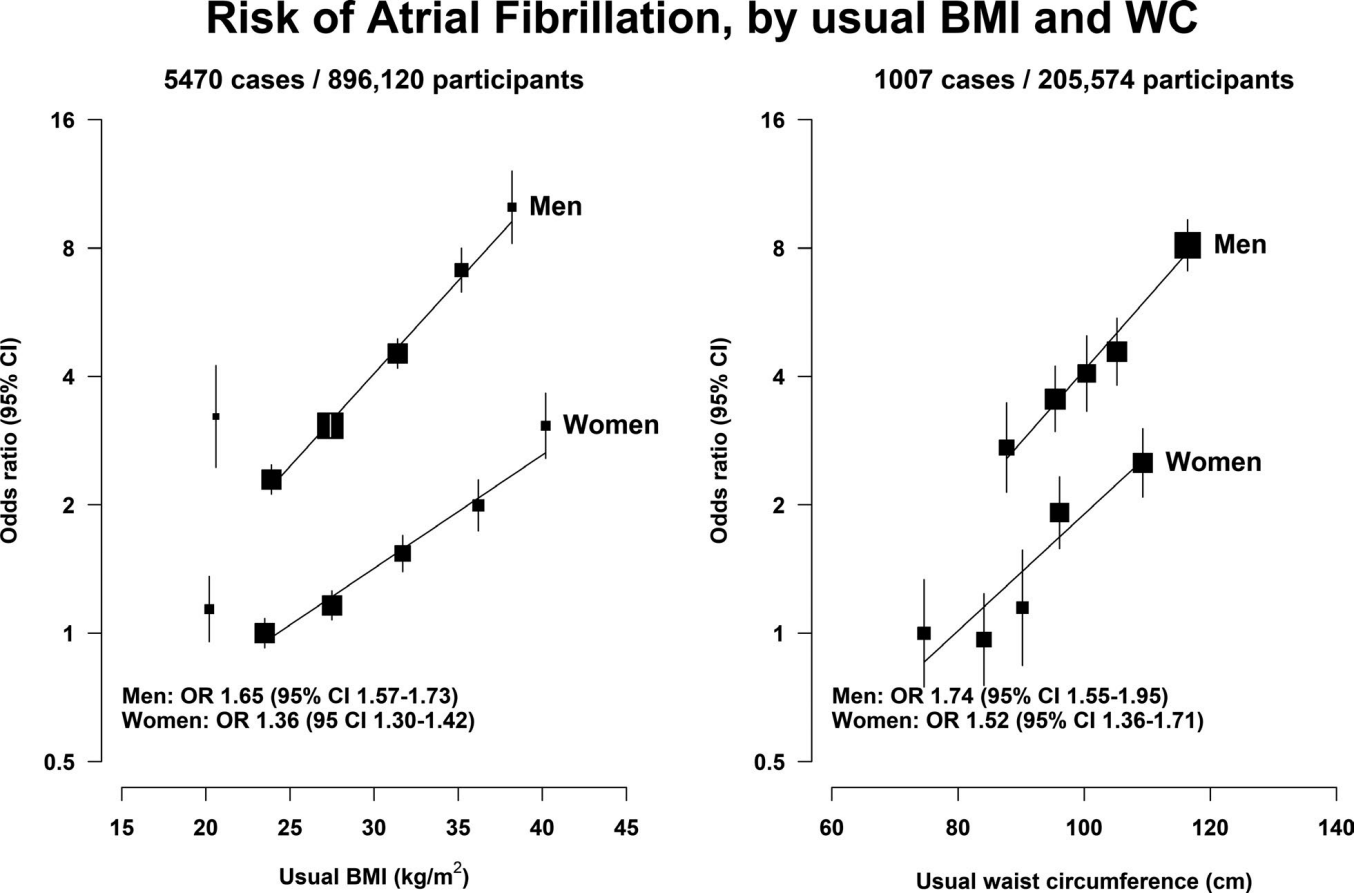
Risk of atrial fibrillation by usual BMI and WC for men and women, using the fully adjusted model. For BMI, women with BMI 20 to 25 kg/m^2^ were used as reference group. For WC, we used the first quintile of WC in women as reference group. ORs of each BMI and WC category were plotted against the mean of the resurvey values (ie, estimated “usual value”). We used group‐specific CIs. The size of the boxes is relative to the total number of participants in each category. The ORs for usual BMI are provided per 5 units increment in participants with BMI ≥20 kg/m^2^. The ORs for usual WC are provided per 14 cm increment for men and 13 cm for women, being the equivalent multiple of the SD of BMI. The number of atrial fibrillation cases and total number of participants per category, the risk estimates, and 95% CI are provided in Table S6 for BMI and Table S7 for WC. BMI indicates body mass index; OR, odds ratio; and WC, waist circumference.

Overall, 0.6% of the participants had AF (n=12 067). The prevalence rose steeply with age and was 2 to 3 times higher in men compared with women for each decade of age (Figure 1). Multivariable analyses of 896 120 participants showed a positive association between usual BMI per 5 kg/m^2^ increment (excluding the lowest BMI group) and AF, with an OR of 1.65 (95% CI, 1.57–1.73) for men and 1.36 (95% CI, 1.30–1.42) for women (*P*_trend_<0.0001). Absolute risks were higher in men compared with women and the relationship was stronger in men (Figure 2 and Table S6). We found a significantly higher risk of AF with higher usual WC in 205 574 participants, with an OR of 1.74 (95% CI, 1.55–1.95) for men per 14 cm increase and 1.52 (95% CI, 1.36–1.71) for women per 13 cm increase (*P*_trend_<0.0001) (Figure 2). Abdominal obesity was also associated with a higher risk of AF, with an OR of 1.83 (95% CI, 1.56–2.15) for men and 1.84 (95% CI, 1.46–2.32) for women when compared with no abdominal obesity (Table S7). We found similar results restricting these analyses to participants in whom both BMI and WC were recorded.

In the analyses of 193 140 participants of the nested sample, there was a stronger improvement of LR χ^2^ for WC than BMI (30% versus 23%, respectively) in men. In contrast, for women BMI showed a stronger improvement of LR χ^2^ than WC (23% versus 12%).

Adding BMI to the fully adjusted models plus WC showed a marginal improvement of LR χ^2^ in men (1%) and showed 9% improvement in women. Adding WC to the fully adjusted models plus BMI showed 6% improvement of LR χ^2^ in men but no improvement in women (Table 2).

**Table 2:** Comparison of Predictive Strengths for Atrial Fibrillation Odds Ratios of Adding Adiposity Measures

	All Participants* (N=193 140)		Men* (N=69 404)		Women* (N=123 736)	
Model (+ Added adiposity measure)	LR χ^2^	Improvement of LR χ^2^ (%)	LR χ^2^	Improvement of LR χ^2^ (%)	LR χ^2^	Improvement of LR χ^2^ (%)
Fully adjusted model without adiposity measures^†^	843.9	…	359.4	…	228.8	…
+ BMI	982.8	139 (16)	443.6	84 (23)	280.5	52 (23)
+ WC	976.3	132 (16)	467.1	108 (30)	256.4	28 (12)
Fully adjusted model with WC^‡^	976.3	…	467.1	…	256.4	…
+ BMI	997.1	21 (2)	469.7	3 (1)	280.6	24 (9)
Fully adjusted model with BMI^§^	982.8	…	443.6	…	280.5	…
+ WC	997.1	14 (1)	469.7	26 (6)	280.6	0 (0)

The χ^2^ value is twice the improvement in the log‐likelihood on addition of extra variables. BMI indicates body mass index; LR, likelihood ratio; and WC, waist circumference.

* Analyses were restricted to 193 140 complete cases in whom both BMI and WC were recorded and with BMI ≥20 kg/m^2^ (nested sample).

^†^ Improvement in LR χ^2^ by the addition of the adiposity measures (either BMI continuous or WC continuous) to the model with full adjustment in which the odds ratio depends on sex (in the analysis of all participants), age groups, country, history of hypertension, diabetes mellitus, smoking status, alcohol use, hypercholesterolemia, and use of antihypertensive medication and lipid‐lowering medication.

^‡^ Improvement in LR χ^2^ by the addition of BMI continuous to the model with WC continuous in which the odds ratio depends on WC, sex (in the analysis of all participants), age groups, country, history of hypertension, diabetes mellitus, smoking status, alcohol use, hypercholesterolemia, and use of antihypertensive medication and lipid‐lowering medication.

^§^ Improvement in LR χ^2^ by the addition of WC continuous to the model with BMI continuous in which the odds ratio depends on BMI, sex (in the analysis of all participants), age groups, country, history of hypertension, diabetes mellitus, smoking status, alcohol use, hypercholesterolemia, and use of antihypertensive medication and lipid‐lowering medication.

**Figure 3:**
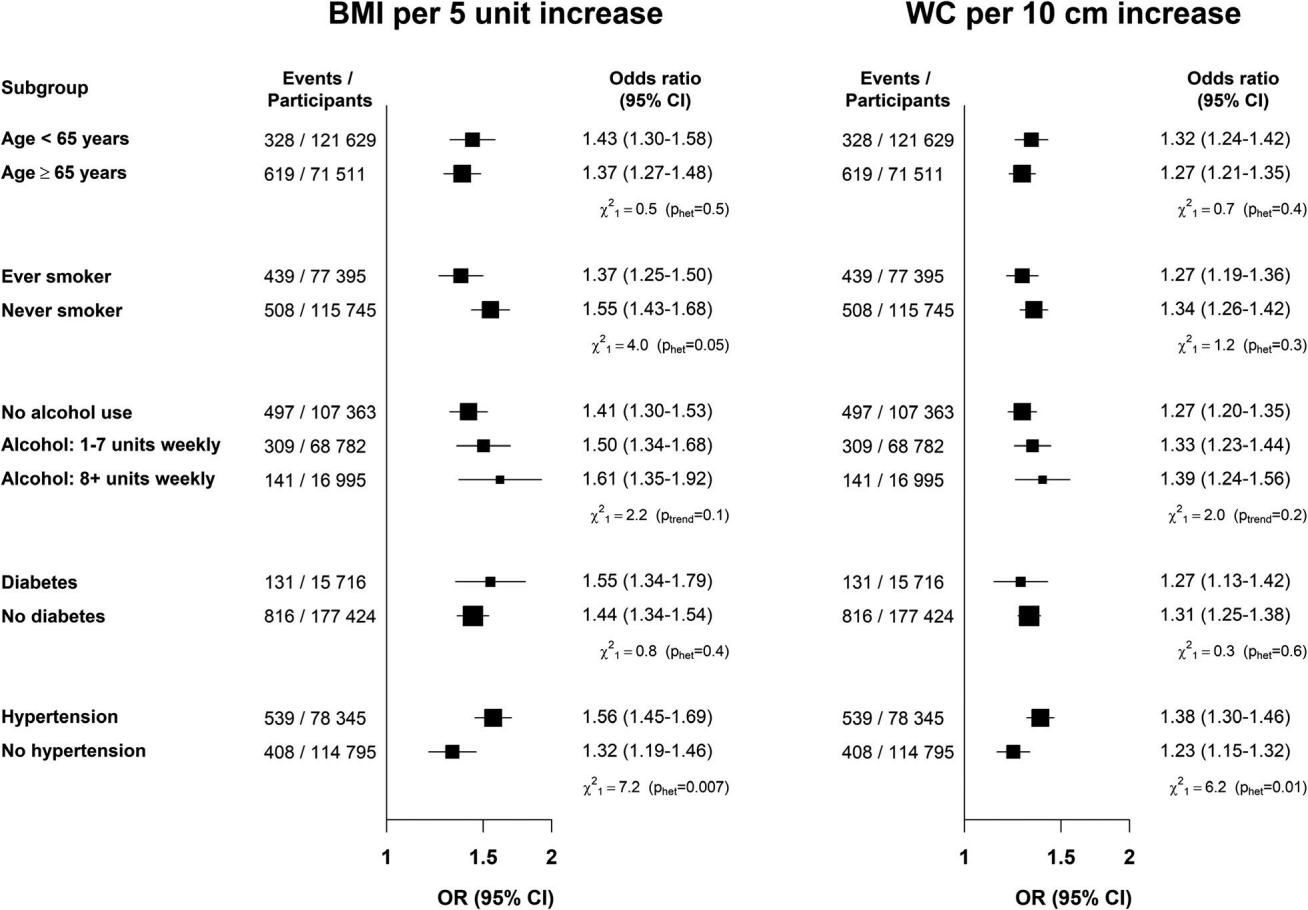
Forest plot of the risk of atrial fibrillation in subgroups, by BMI and WC. Forest plot showing the risk of atrial fibrillation in subgroups, by BMI and WC. Analyses were restricted to 193 140 participants in complete cases in whom both BMI and WC were recorded and with BMI ≥20 kg/m^2^ and without missing values of covariates included in the multivariable model with full adjustment (nested sample). The ORs for BMI are shown per 5 units increment and the ORs for WC are shown for an increase of 10 cm. Ever smoker was defined as either current or former smoker. Hypertension was defined as either a reported history of hypertension or use of antihypertensive therapy. BMI indicates body mass index; OR, odds ratio; and WC, waist circumference.

Subgroup analyses found consistent results across age, smoking status, alcohol use, and reported history of diabetes mellitus. The positive association of both BMI and WC with the risk of AF was higher in participants with reported hypertension or use of antihypertensive therapy compared with no reported hypertension/antihypertensive therapy (*P*_het_=0.007 and *P*_het_=0.01, respectively) (Figure 3).

## Discussion

In this large cross‐sectional study, including over 2 million screened participants, we found a positive log‐linear association between BMI (except for the lowest BMI group) and WC and the risk of AF. We found higher risks of AF in men than women. BMI is more informative about risk of AF in women whereas WC is more informative in men.

The risk of AF is higher in men compared with women, but the difference in AF incidence attenuates in older patients aged 80 and above.^22^ Reasons for these differences include sex‐specific atrial electrophysiologic properties, atrial remodeling, and mechanisms of atrial fibrosis. BMI has been identified as a risk factor for AF. A recent meta‐analysis including 25 studies found a nonlinear relationship between BMI and AF risk, with higher BMI values associated with a steeper increase in risk.^23^ In their meta‐analysis, a 5‐unit increment in BMI was associated with a 28% increased relative risk of AF (RR, 1.28; 95% CI, 1.20–1.38).^23^ Their subgroup analysis showed a stronger association in men compared with women, with an RR of 1.39 (95% CI, 1.30–1.48) for men compared with 1.30 (95% CI, 1.14–1.48) for women.

WC has previously been shown to provide additional predictive information on all‐cause mortality beyond BMI.^24^ Only a limited number of studies have looked at the association between WC and AF risk.^10^, ^11^, ^12^, ^13^, ^14^ When pooled in a meta‐analysis, these results appeared to show a roughly linear relationship with a summary risk ratio for a 10 cm increase in WC of 1.18 (95% CI, 1.12–1.25).^23^ Two studies that provided risk estimates by sex showed that the risk in men seems higher than women.^10^, ^13^ In addition, we found that BMI is more informative about risk of AF in women, whereas WC is more informative in men.

### Strengths and Limitations

Our study is one of the largest to date to assess the association between adiposity measures and AF. We were able to compare BMI, WC, and their association with AF both individually and in combination and we determined sex‐specific analyses. We adjusted for regression dilution bias and excluded participants with cardiovascular and cardiac disease to minimize the risk of reverse causation. Standardized measurement of outcome was used, including a 12‐lead ECG to confirm a diagnosis of AF, reviewed by physicians who received in‐house training.

Using single time point ECG is likely to underestimate the true prevalence of AF in the population, as cases of paroxysmal and persistent AF may be missed and were consequently included in the "no AF" group. This might have contributed to a lower prevalence of AF compared with other populations. Other reasons might be the inclusion of relatively young participants and a high proportion of female participants in our study as well as the exclusion of participants with cardiovascular disease. The prevalence was, however, comparable with the prevalence of 0.5% found in the STROKESTOP study that included participants aged 75 to 76 years.^25^ We were not able to validate the diagnosis of AF and reported comorbidities, for example via health records. Similarly, there may have been confounding factors missed that contribute to the observed relationship between underweight and increased AF risk, such as muscle wasting conditions or hyperthyroidism. Participants were self‐referred and self‐funded, which might influence generalizability to other populations. However, relative measures (associations with risk factors) tend to be less affected by selection bias.^26^ Recall bias should be considered for characteristics that were self‐reported. The type of antihypertensive agent was not recorded. BMI was based on self‐reported weight and height, but reporting errors might not affect suitability for analyses.^16^ However, others found that the accuracy of self‐reported height and weight was different for men and women.^27^ WC was available in a subset of participants but we performed comparative analyses in the subset of participants in whom both BMI and WC were recorded (Table 2). Relying on BMI and WC may not fully account for differences in proportion of muscle mass and adipose tissue. The number of participants with resurvey measurement was small and this might affect the preciseness of the correction for regression dilution, and this number was too small to perform analysis of change in measures of adiposity and risk of AF. Sensitivity analyses showed that the shape of the associations between BMI and WC and the risk of AF was constant across levels of the confounders, but collapsibility bias is always possible with logistic regression. The cross‐sectional study design may underestimate the importance of previous weight change as obesity in early life appears to confer a long‐term increase in risk of AF even after accounting for subsequent weight loss.^28^

### Implications for Practice

Our cross‐sectional data highlight the important relationship between increasing weight and AF risk and the difference in informativeness of adiposity measures in men and women. When assessing adiposity measures in clinical practice, WC might be a more informative measure about risk of AF in men and BMI in women. This stresses the importance of sex‐specific risk prediction of AF.^29^ Longitudinal data showed weight gain over time increases the risk of AF, irrespective of baseline weight status and sex.^30^ Among 15 214 participants in the HUNT (Nord‐Trøndelag Health Study), overweight and obesity were associated with an increased risk of AF compared with healthy weight, but so too was both weight loss and weight gain over a median of 8 years follow‐up when compared with people with stable weight.^28^ Interventions to prevent weight gain and promote healthy weight might therefore help reduce the burden of AF in the population.

The LEGACY (Long‐Term Effect of Goal‐Directed Weight Management on Atrial Fibrillation Cohort: A 5‐Year Follow‐Up Study) randomized controlled trial demonstrated that intentional weight loss through a goal‐directed weight management program could help reduce AF symptom burden in people who were overweight at baseline.^31^ However, as yet there is no consistent evidence that nonsurgical weight loss leads to a reduction in AF incidence.^32^ Although weight reduction in overweight or obese individuals is likely to have cardiovascular benefits beyond the risk of AF, the current evidence base supports public health strategies that promote maintenance of a healthy weight. Further research is needed to confirm the sex‐specific associations between adiposity measures and AF risk so that interventions can be targeted at appropriate populations and risk prediction of AF should consider sex‐specific differences.

## Conclusions

Our study highlights the importance of overweight and obesity as potentially modifiable AF risk factors. BMI may be a more informative measure of AF risk in women and WC in men. This stresses the importance of sex‐specific risk prediction of AF. Clinicians should consider measuring and addressing adiposity where possible. Interventional studies are required to demonstrate whether intentional weight loss can reduce the risk of AF. At present public health strategies and health promotion should advise individuals to maintain a healthy weight and avoid weight gain.

## Sources of Funding

Professor Halliday is funded by the UK Health Research (NIHR) Oxford Biomedical Research Centre (BRC). Sarah Lewington is funded by the UK Medical Research Council and the CDC foundation (with support from Amgen). The study funders had no role in study design; data collection, analysis, or interpretation; or drafting of the report. The corresponding author had full access to all data in the study and had final responsibility for the decision to publish the report.

## Disclosures

Nicholas R Jones reports support from a Wellcome Trust Doctoral Research Fellowship grant (grant number 203921/Z/16/Z). Sarah Lewington reports grants from UK Medical Research Council during the conduct of the study; grants from CDC Foundation outside the submitted work. Richard Bulbulia reports grants from UK Medical Research Council during the conduct of the study; grants from UK Medical Research Council outside the submitted work. Alison Halliday is funded by the UK Health Research (NIHR) Oxford Biomedical Research Centre (BRC). The remaining authors have no disclosures to report.

## Supporting information


Table S1–S7
Figure S1
