# Supplementary material for: Joint Associations Between Body Mass Index and Waist Circumference With Atrial Fibrillation in Men and Women
Source: J Am Heart Assoc. 2021 Apr 15;10(8):e019025. doi: 10.1161/JAHA.120.019025 (PMC8174185; doi:10.1161/JAHA.120.019025)

SUPPLEMENTAL MATERIAL

Table S1. STROBE checklist for cross-sectional studies.

Section/Topic	Item	Checklist Item	Page
**Title and abstract**			
	1	(a) Indicate the study's design with a commonly used term in the title or the abstract<br>(b) Provide in the abstract an informative and balanced summary of what was done and what was found	✓ Title & Abstract
**Introduction**			
Background/ rationale	2	Explain the scientific background and rationale for the investigation being reported	✓ Intro
Objectives	3	State specific objectives, including any prespecified hypotheses	✓ Intro
**Methods**			
Study design	4	Present key elements of study design early in the paper	✓ M&M
Setting	5	Describe the setting, locations, and relevant dates, including periods of recruitment, exposure, follow-up, and data collection	✓ M&M
Participants	6	(a) Give the eligibility criteria, and the sources and methods of selection of participants	✓ M&M
Variables	7	Clearly define all outcomes, exposures, predictors, potential confounders, and effect modifiers. Give diagnostic criteria, if applicable	✓ M&M
Data sources/ measurement	8*	For each variable of interest, give sources of data and details of methods of assessment (measurement). Describe comparability of assessment methods if there is more than one group	✓ M&M
Bias	9	Describe any efforts to address potential sources of bias	✓ M&M
Study size	10	Explain how the study size was arrived at	✓ M&M
Quantitative variables	11	Explain how quantitative variables were handled in the analyses. If applicable, describe which groupings were chosen and why	✓ M&M
Statistical methods	12	(a) Describe all statistical methods, including those used to control for confounding	✓ M&M
		(b) Describe any methods used to examine subgroups and interactions	
		(c) Explain how missing data were addressed	
		(d) If applicable, describe analytical methods taking account of sampling strategy	
		(e) Describe any sensitivity analyses	
**Results**			
Participants	13*	(a) Report numbers of individuals at each stage of study—eg numbers potentially eligible, examined for eligibility, confirmed eligible, included in the study, completing follow-up, and analysed	✓ M&M
		(b) Give reasons for non-participation at each stage	✓ Results
		(c) Consider use of a flow diagram	NA
Descriptive data	14*	(a) Give characteristics of study participants (eg demographic, clinical, social) and information on exposures and potential confounders	✓ Results
		(b) Indicate number of participants with missing data for each variable of interest	✓ Table 1
Outcome data	15*	Report numbers of outcome events or summary measures	✓ Table 1
Main results	16	(a) Give unadjusted estimates and, if applicable, confounder-adjusted estimates and their precision (eg, 95% confidence interval). Make clear which confounders were adjusted for and why they were included	✓ Results
		(b) Report category boundaries when continuous variables were categorized	
		(c) If relevant, consider translating estimates of relative risk into absolute risk for a meaningful time period	
Other analyses	17	Report other analyses done—eg analyses of subgroups and interactions, and sensitivity analyses	✓ Results & Table 3
**Discussion**			
Key results	18	Summarise key results with reference to study objectives	✓ Discussion
Limitations	19	Discuss limitations of the study, taking into account sources of potential bias or imprecision. Discuss both direction and magnitude of any potential bias	✓ Discussion

Interpretation	20	Give a cautious overall interpretation of results considering objectives, limitations, multiplicity of analyses, results from similar studies, and other relevant evidence	✓ Discussion
Generalisability	21	Discuss the generalisability (external validity) of the study results	✓ Discussion
**Other information**			
Funding	22	Give the source of funding and the role of the funders for the present study.	✓

*Give information separately for exposed and unexposed groups.

Table S2. Number of excluded participants, with reasons for exclusion.

**Reason for exclusion**	**Number of participants***
Reported history of CVD (CHD, stroke or TIA, PAD)	336,339
Reported history of congestive heart failure	20,591
Reported history of valvular disease or left ventricular hypertrophy	89,844
Reported history of COPD	64,275
No ECG	332,195
BMI and WC not recorded	82,602
Missing sex	11,431
Missing smoking history	250,134
**Total number included in our study**	**2,088,728**

* Sequential exclusion.

CVD indicates cardiovascular disease; CHD, coronary heart disease; COPD, chronic obstructive pulmonary disease; ECG, electrocardiogram; PAD, peripheral arterial disease; TIA, transient ischemic attack.

Table S3. Missing data in our cohort.

Variable	Percentage of participants with missing
Age	0
Sex	0
Smoking status	0
Height	0.2
BMI	0.5
Hypertension or antihypertensive therapy	7.5
Hypercholesterolemia or lipid-lowering therapy	7.8
Diabetes	8.3
Alcohol use	55.0
Waist circumference	85.7

BMI, body mass index.

Table S4. Overview of regression dilution ratios.

Exposure	Analysis	Spearman regression dilution ratio
BMI	In men	0.86
	In women	0.89
WC	In men	0.79
	In women	0.82

BMI indicates body mass index; WC, waist circumference

Table S5. Baseline characteristics in participants with both BMI and WC recorded.

	Participants with AF (n = 1430)	Participants without AF (n = 287,951)	All Participants (n = 289,381)
Age (y)	67.8 ± 8.7	61.0 ± 9.3	61.0 ± 9.3
Female sex	517 (36.2)	184,039 (63.9)	184,556 (63.8)
Height in males (m)	1.80 ± 0.1	1.78 ± 0.1	1.78 ± 0.1
Height in females (m)	1.64 ± 0.1	1.63 ± 0.1	1.63 ± 0.1
BMI (kg/m^2)	29.9 ± 5.9	28.2 ± 5.4	28.2 ± 5.4
WC (cm)	102.5 ± 16.3	93.9 ± 15.0	93.9 ± 15.1
Male ever smoker[1]	473 (51.8)	46,414 (44.7)	46,887 (44.7)
Female ever smoker[1]	190 (36.8)	69,364 (37.7)	69,554 (37.7)
Current alcohol use	494 (48.3)	90,790 (44.4)	91,284 (44.4)
Hypertension or antihypertensive medication	774 (56.9)	108,738 (38.7)	109,512 (38.8)
Diabetes mellitus	179 (13.4)	20,541 (7.5)	20,720 (7.5)
Hypercholesterolemia or lipid-lowering medication	672 (49.2)	126,048 (45)	126,720 (45)
Creatinine (mg/dL)	0.9 ± 0.3	0.8 ± 0.2	0.8 ± 0.2

Values are mean ± SD for continuous variables and n (%) for categorical variables.
AF indicates atrial fibrillation; BMI, body mass index; SD, standard deviation; WC, waist circumference.
[1] Ever smoker was defined as current or former smoker.

Table S6. Odds ratios of AF by BMI in men and women.

	Women			Men		
	Number of events / female participants	Mean usual BMI	OR (95% CI)	Number of events / male participants	Mean usual BMI	OR (95% CI)[1]
BMI category[2]						
<20 kg/m^2	114 / 26,493	20.2	1.14 (0.95-1.37)	54 / 4406	20.6	3.23 (2.46-4.23)
20-<25 kg/m^2	644 / 191,149	23.5	1.00 (0.92-1.08)	551 / 61,563	23.9	2.29 (2.10-2.50)
25-<30 kg/m^2	755 / 203,806	27.5	1.16 (1.08-1.24)	1365 / 143,554	27.4	3.08 (2.91-3.25)
30-<35 kg/m^2	489 / 110,304	31.7	1.54 (1.41-1.69)	741 / 64,977	31.4	4.52 (4.20-4.87)
35-<40 kg/m^2	213 / 43,295	36.2	2.00 (1.74-2.29)	293 / 18,829	35.2	7.11 (6.31-8.00)
≥40 kg/m^2	138 / 21,569	40.2	3.08 (2.60-3.65)	113 / 6175	38.2	10.01 (8.28-12.11)
**Total**	**2353 / 596,616**	**-**	**-**	**3117 / 299,504**	**-**	**-**
Trend test (in participants with BMI ≥20 kg/m^2)			*$X^2(1)=194.99$ $P<0.0001$*			*$X^2(1)=407.78$ $P<0.0001$*
**Usual BMI per 5 units increment (in participants with BMI ≥20 kg/m^2)**	**2239 / 570,123**	**-**	**1.36 (1.30-1.42)**	**3063 / 295,098**	**-**	**1.65 (1.57-1.73)**

Model with full adjustment for adjustment for age groups, country, history of hypertension, diabetes, smoking status, alcohol use, hypercholesterolemia, use of anti-hypertensive medication and lipid-lowering medication. We used group-specific confidence intervals.

BMI indicates body mass index; CI, confidence interval; OR, odds ratio.

[1] BMI 20-<25 kg/m^2 in women was as reference category. [2] BMI was categorized according to baseline BMI values.

Table S7. Odds ratios of AF by WC in men and women.

	Women			Men		
	Number of events / female participants	Mean usual WC	OR (95% CI)[1]	Number of events / male participants	Mean usual WC	OR (95% CI)
WC category[2]						
WC quintile 1	47 / 27,540	74.6	1.00 (0.75-1.34)	68 / 14,506	87.7	2.72 (2.14-3.47)
WC quintile 2	64 / 35,261	84.1	0.97 (0.76-1.24)	128 / 18,797	95.4	3.54 (2.97-4.22)
WC quintile 3	40 / 17,871	90.2	1.15 (0.84-1.57)	95 / 11,858	100.4	4.06 (3.32-4.98)
WC quintile 4	106 / 27,722	96.1	1.92 (1.58-2.33)	120 / 13,089	105.2	4.57 (3.81-5.48)
WC quintile 5	119 / 24,838	109.4	2.51 (2.08-3.02)	220 / 14,092	116.4	8.12 (7.07-9.32)
**Total**	**376 / 133,232**	**-**	-	**631 / 72,342**	**-**	-
Trend test			$X^2(1)=50.08$ $P<0.0001$			$X^2(1)=89.60$ $P<0.0001$
**Usual WC per 13 cm increment**[3]	**376 / 133,232**	-	**1.52 (1.36-1.71)**	-	-	-
**Usual WC per 14 cm increment**[3]	-	-	-	**631 / 72,342**	-	**1.74 (1.55-1.95)**
**Baseline abdominal obesity vs. not**[4]	**376 / 133,232**	-	**1.84 (1.46-2.32)**	**631 / 72,342**	-	**1.83 (1.56-2.15)**

Model with full adjustment for age groups, country, history of hypertension, diabetes, smoking status, alcohol use, hypercholesterolemia, use of anti-hypertensive medication and lipid-lowering medication. We used group-specific confidence intervals.

CI indicates confidence interval; OR, odds ratio; WC, waist circumference.

[1] WC quartile 1 in women was as reference category.

[2] Quintiles were categorized according to baseline WC values. In men, quintiles were <89, 89-97, 97-102, 102-109, and >109 cm. In women, quintiles were < 76, 76-86, 86-91, 91-102, and >102 cm.

[3] Usual WC was calculated per 14 cm increment in men and 13 cm in women, since these are an equivalent multiple of the standard deviation as BMI. For men, the SD of BMI was 4.6 and of WC was 13.2. The WC OR for men is calculated for a change of $5 \div 4.6 \times 13.2 = 14\ cm$. For women, the SD of BMI was 5.6 and of WC was 15.1. The WC OR for women is calculated for a change of $5 \div 5.6 \times 15.1\ = 13\ cm$.

[4] Abdominal obesity was defined as WC of >102 cm in men or >88 cm in women.

Figure S1. Flow diagram of patients included in the analysis.

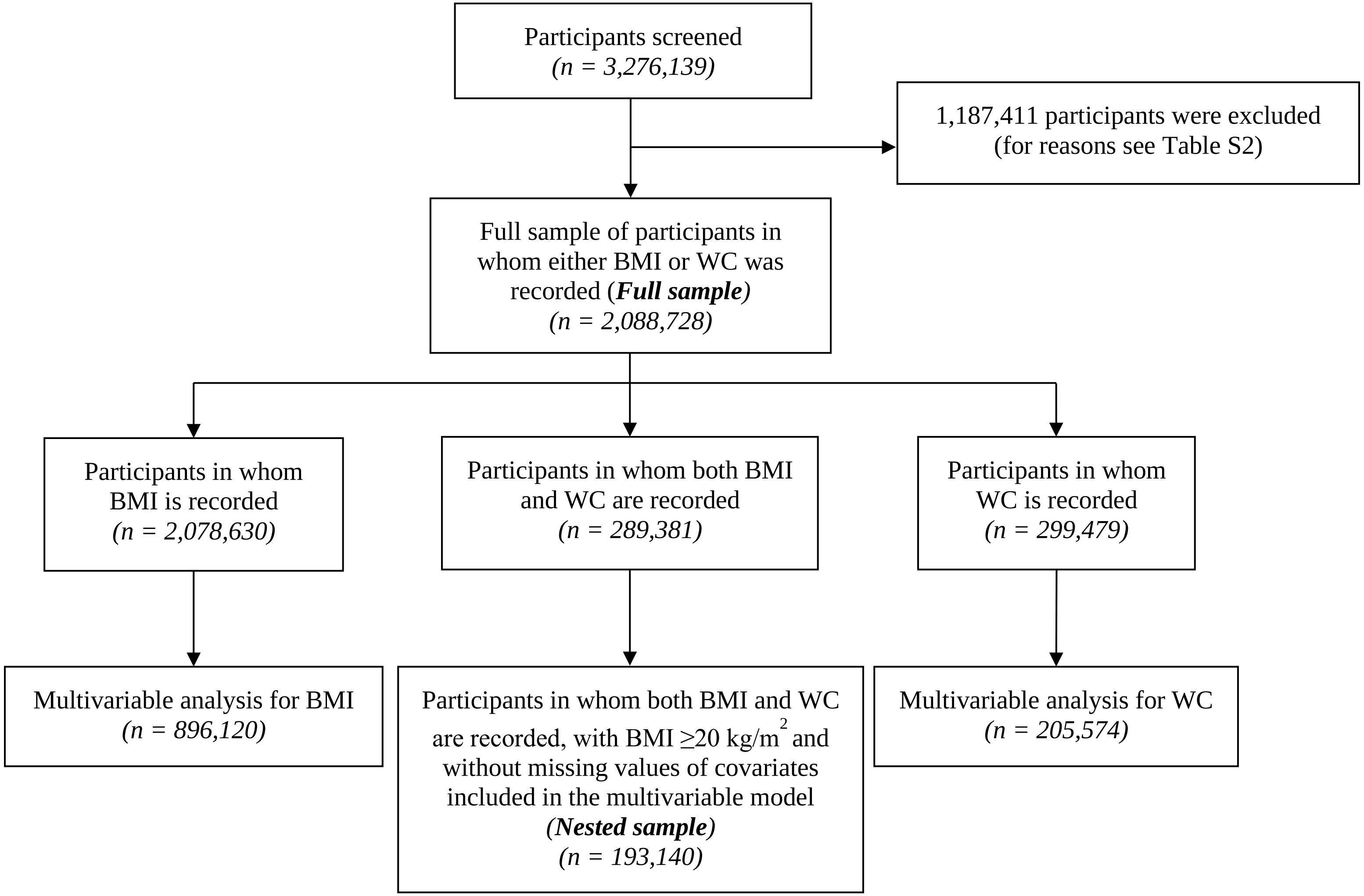

Supplement: Supplementary file 1 — Table S1–S7 Figure S1 [file JAH3-10-e019025-s001.pdf]
